# Integrating Physical Therapy and Virtual Reality to Manage Pain-Related Fear of Movement in Patients With Chronic Pain: A Randomized Controlled Trial

**DOI:** 10.7759/cureus.79551

**Published:** 2025-02-24

**Authors:** Shunsuke Sakuma, Kazuhiro Kimpara, Yosuke Kawai, Yorihide Yanagita, Natsumi Tanaka, Yuichi Tawara, Genichiro Matsui, Kazuhiro Terada, Shinichi Arizono

**Affiliations:** 1 Department of Rehabilitation Medicine, Omaezaki Pain Clinic, Omaezaki, JPN; 2 School of Rehabilitation Sciences, Seirei Christopher University, Hamamatsu, JPN; 3 Department of Rehabilitation Medicine, Terada Pain Clinic, Hamamatsu, JPN; 4 Department of Physical Therapy Science, Nagasaki University Graduate School of Biomedical Sciences, Nagasaki, JPN

**Keywords:** chronic pain, kinesiophobia, pain-related fear of movement, physical therapy, virtual reality

## Abstract

Objectives: This study aims to investigate the effect of virtual reality (VR) technology and physical therapy on pain-related fear of movement in patients with chronic pain.

Methods: This randomized controlled trial included 73 patients with chronic pain. All participants underwent measurements using the Tampa Scale for Kinesiophobia (TSK), the Japanese version of the World Health Organization Health and Work Performance Questionnaire (WHO-HPQ), the Pain Self-Efficacy Questionnaire (PSEQ), the Numerical Rating Scale (NRS), the Hospital Anxiety and Depression Scale (HADS), the Pain Catastrophizing Scale (PCS), the International Physical Activity Questionnaire (IPAQ), the EuroQOL 5 Dimensions 5-Level (EQ-5D-5L), quadriceps muscle strength, the Timed Up and Go test (TUG), and the 30-s Chair Stand test (CS-30). The intervention group underwent three months of rehabilitation, incorporating VR and exercises administered by a physical therapist. The control group performed similar exercises guided by a physical therapist, with the only distinction being the absence of VR.

Results: Sixty-six participants (32 males, mean age: 54.7±11.7 years) were included in the analysis. TSK scores improved at one-month post-intervention in the intervention group (ΔTSK: -12.4±2.1) compared to the control group (ΔTSK: -7.8±1.4) (p<0.05). The intervention group predominantly demonstrated improved TSK scores at one-month post-intervention. Multivariate logistic regression analysis at one-month post-intervention revealed significant associations between TSK scores and the intervention, PCS, PSEQ, and IPAQ scores (p<0.05).

Discussion: This study revealed two significant findings: (i) combining physical therapy with VR effectively reduced pain-related fear of movement in patients at an early stage, and (ii) a change in the kinesiophobia scores in patients with chronic pain was associated with a change in pain catastrophizing, self-efficacy, and VR intervention. TSK scores in the intervention group significantly improved at one-month post-intervention compared to those in the control group. Combining VR with physical therapy effectively mitigated the excessive pain-related fear of movement. In this study, VR provided an exercise experience without physical pain for patients with chronic pain. This was intended to create a calming, distracting environment and thereby reduce the perceived threat of kinesiophobia. Furthermore, it is possible that there was a reduction in central sensitization. Multiple regression analysis revealed that ΔPCS, ΔPSEQ, and intervention were common elements of ΔTSK at one and three months post-intervention. These findings suggest that combined physical therapy with VR and improved PCS and PSEQ scores contributed to improved TSK scores. Therefore, VR could be beneficial in managing pain-related fear of movement by creating the illusion that exercise does not cause pain. This study had several limitations. First, pain assessment was subjective using questionnaires. Second, participants were limited to patients who underwent physical therapy, which may introduce selection bias. Third, findings were derived from a single pain clinic.

Conclusion: This study’s findings indicated significant differences between the control and intervention groups, which combined physical therapy with VR, particularly in reducing pain-related fear of movement at an early stage.

## Introduction

Pain-related fear of movement, characterized by the dominant belief that pain and reinjury are the likely consequences of movement, can maintain and exacerbate disability associated with chronic pain [1]. Prolonged pain-related fear of movement can lead to negative thoughts and beliefs. Negative thoughts and beliefs about pain can lead patients to engage in pain-avoidance behaviors, resulting in inactivity and hindering recovery and pain reduction [2]. For patients with chronic pain, the consequences of disuse and reduced activity can be severe, increasing the risk of a wide range of health problems, functional decline, and premature death [2]. Furthermore, pain-related fear of movement has been identified as a factor that decreases activities of daily living (ADL) and quality of life (QOL) in patients with chronic pain and also adversely affects job performance [3].

Virtual reality (VR) has recently garnered attention as a pain management tool [4]. VR treatment serves as an effective pain distraction mechanism by focusing on external stimuli rather than body movements, thereby reducing attention to pain through divided attention tasks [4]. Previous research suggests that the therapeutic mechanisms of VR in treating chronic low back pain are unclear but may include distraction, neuromodulation of body perception, and graded exposure therapy [5]. VR has demonstrated efficacy and feasibility in delivering pain management skills, including distraction and exposure therapies [5]. Furthermore, compared to traditional methods, VR is considered a cost-effective and efficient tool. A systematic review has explored the effects of VR on patients with chronic low back pain [6]. This review identified evidence that VR positively impacts pain, function, mobility, functional capacity, psychological outcomes, QOL, neuropsychological outcomes, and bodily sensations in patients with chronic pain. Additionally, a previous systematic review has shown that VR significantly diminishes pain intensity and pain-related fear of movement in patients with chronic low back pain [7]. Recent studies have shown that VR intervention involving movement or game-like elements can improve pain and reduce pain-related fear of movement [8]. In a previous study, an RCT involving 72 patients with chronic low back pain reported that the group that underwent treatment combined with VR showed a significant reduction in pain-related fear of movement and pain intensity at five-month follow-up [8]. While exercise therapy is an important treatment for chronic pain, pain-related fear of movement may act as a hindering factor. Therefore, patients with chronic pain and a pain-related fear of movement need targeted treatment for this issue.

Many VR systems are currently used to manage acute pain [4], but their clinical application in the chronic phase also needs to be considered. A problem with chronic pain is a pain-related fear of movement [1], and while exercise therapy is an important treatment for chronic pain, pain-related fear of movement can be an inhibiting factor. Although the effectiveness of VR has been verified for patients with chronic low back pain [5], the evidence is still lacking. Furthermore, it needs to be verified for patients with chronic pain in general. We speculate that for chronic pain patients, VR may be effective against pain-related fear of movement.

The novelty of this study is that it shows that a combined VR and physical therapy treatment is effective in reducing pain-related fear of movement in patients with chronic pain. Therefore, this study aimed to investigate the effects of VR technology and physical therapy on pain-related fear of movement in patients with chronic pain.

## Materials and methods

Study design

This randomized controlled trial was conducted in accordance with the Consolidated Standards of Reporting Trials statement. The purpose and protocol of the study were explained to all participants, and written informed consent was obtained. Data collection occurred from April 2022 to March 2023.

Participants

The participants were patients with chronic musculoskeletal pain who visited the Terada Pain Clinic in Japan. The inclusion criteria were (a) patients experiencing chronic pain for at least three months, (b) age 18-75 years, and (c) current employment. The exclusion criteria were: (a) evident acute pain, such as trauma; (b) severe cognitive impairment (e.g., patients who are unable to carry out ADL independently or have difficulty communicating during routine medical care); (c) inability to provide informed consent; and (d) patients with symptoms incompatible with VR (e.g., motion sickness, dizziness, or vestibular symptoms). Ethical approval was obtained from Seirei Christopher University’s Ethics Committee (approval number 21043). The study was registered in the UMIN Clinical Trials Registry (UMINID: 000051194). All procedures adhered to the ethical standards of the Declaration of Helsinki [9]. Additionally, a detailed explanation of the study procedures was provided to all participants who signed a consent form before enrollment.

Randomization and blinding

Participants were randomized into intervention and control groups using computerized random numbers. The randomization schedule was computer-generated through random permuted blocks of sizes 6-8, executed by a researcher not involved in recruiting participants. Access to the randomization schedule was restricted to a password-protected computer program. Participants were blinded to the study hypotheses and informed that a comparison would be made between two programs designed for persons with chronic pain. Following randomization, participants were provided with only the details of the exercise program they were to undertake. Patients were blinded to the interventions in the other groups.

Intervention group

The intervention group underwent a three-month rehabilitation program, which combined VR and exercise sessions administered by a physical therapist. The primary aims of the VR and exercise interventions were to reduce pain and pain-related fear of movement. The physical therapist was informed about the group’s assignment. Participants commenced the VR and exercise program after physical and psychological assessments. The VR therapy was conducted at the beginning of each session.

The VR system employed in this study was the 128 GB Oculus Quest® (Metaverse Inc., USA), a standalone headset. The VR content used in this study featured an immersive experience simulating a walk through a forest. Participants were seated in a chair and wore the VR headset during a 10-minute video session. During the session, participants felt like they were walking in the woods. In this study, the intervention was carried out once a week for three months, totaling 12 sessions.

The primary aim of the exercises was to decrease pain and improve physical activity. The exercise program was conducted with modifications based on previous studies [10]. After undergoing a physical assessment, participants commenced 20 minutes of stretching and muscle-strengthening exercises, followed by approximately 20 minutes of ergometer or treadmill exercises. The exercise program was carried out for three months, once or twice a week, for 40 minutes per session. Each stretch was performed in three sets of 30-second holds for each muscle group, with participants encouraged to enhance the stretch range over the treatment period. For example, participants with neck pain were given trapezius muscle stretches, and participants with low back pain were given gluteus maximus muscle stretches. Stretching exercises were also recommended for participants experiencing pain in other areas. The intensity and frequency of the exercises were progressively increased during the intervention period (exercise intensity was low-moderate). The exercise program was conducted in stages, beginning with pain-free areas and paying close attention to each patient’s fear of exercise. Participants were instructed to perform home exercises. The home exercises included stretching for joints or muscles with reduced flexibility, muscle strengthening exercises, and 60 minutes of aerobic exercise per day. These exercises were designed to be performed with ample rest. A physical therapist checked compliance with the home exercises during each intervention. Notably, if participants reported excessive pain during an exercise, the exercise was stopped, or the intensity was temporarily reduced. The physical therapist then addressed the pain at the next intervention.

Control group

The control group performed the same exercises as the intervention group and received treatment from a physical therapist. The sole difference between the control and intervention groups was the absence of VR treatment.

Outcome measurements

All participants underwent measurements using the Tampa Scale for Kinesiophobia (TSK), the Japanese version of the World Health Organization Health and Work Performance Questionnaire (WHO-HPQ), the Pain Self-Efficacy Questionnaire (PSEQ), the Numerical Rating Scale (NRS), the Hospital Anxiety and Depression Scale (HADS), the Pain Catastrophizing Scale (PCS), the International Physical Activity Questionnaire (IPAQ), the EuroQOL 5 Dimensions 5-Level (EQ-5D-5L), quadriceps muscle strength, the Timed Up and Go test (TUG), and the 30-s Chair Stand test (CS-30). A physical therapist conducted all outcome measurements. Chronic pain causes not only physical problems but also various other problems such as psychological problems and social problems. Therefore, it is necessary to understand the patient's condition based on a multifaceted evaluation of the evaluation tool. The primary outcome was TSK. The secondary outcomes were WHO-HPQ and PSEQ. All outcome measurements were conducted three times: at the initial stage, one month after intervention, and three months after intervention.

The TSK assesses fear of movement or reinjury [1]. It has demonstrated adequate reliability, parallel-criterion-related validity, and incremental validity [11]. The total TSK score ranges from 17 to 68, with 17 indicating no kinesiophobia, 68 indicating moderate kinesiophobia, and 37 indicating the presence of kinesiophobia.

The Japanese version of the WHO-HPQ was used to assess absolute and relative presenteeism [12]. This reliable and valid self-report questionnaire assesses how health problems interfere with an individual’s ability to perform job tasks.

The PSEQ was used to assess patients’ self-efficacy in pain management; higher scores indicated greater self-efficacy and functioning despite pain [13].

Pain intensity was assessed using NRS scores, where 0 indicated no pain, and 10 indicated the highest possible degree of pain [14]. Pain intensity was evaluated as the average, minimum, and maximum values in the previous seven days.

The HADS is a short self-assessment questionnaire that measures anxiety and depression. It comprises seven items each for the HADS-depression and HADS-anxiety scales. The HADS is frequently used in both clinical practice and research, exhibiting good psychometric characteristics [15].

The PCS comprises 13 items rated on a five-point Likert scale [16]. This scale measures three dimensions of catastrophizing: rumination, helplessness, and magnification. Respondents are asked to reflect on their past painful experiences and rate the degree to which they have experienced negative thoughts or feelings about pain.

Physical activity levels were measured using the IPAQ. Physical activity was documented in terms of intensity (walking, moderate, or vigorous), frequency, and duration of daily activities. The results from each category of physical activity (walking + moderate-intensity + vigorous-intensity) were summed to obtain the total physical activity in METs/minutes/week [17].

The EQ-5D-5L is an instrument that standardizes various diseases and serves as a complementary assessment tool to existing health-related QOL measures [18]. The Japanese version of the EQ-5D-5L was used, and a Japanese scoring system was established, demonstrating validity and reliability [18].

The maximum isometric knee extensor strength of the legs was measured using a handheld dynamometer (Mobie, Sakai Medical Co., Ltd., Japan). Measurements were performed using a method previously validated for community-dwelling older fallers [19]. The Mobie is a tool used to objectively quantify muscle strength in clinical practice. The maximum force was recorded in kilograms (kgf), and two measurements were taken for each leg [19]. Participants were instructed to remain seated in an upright position. The knee was positioned at 90° flexion, with the Mobie attached 10 cm proximal to the lateral malleolus and secured in place with an inelastic strap looped around the therapy bed and fastened. The strap length allowed isometric contraction to be performed with the knee at 90° flexion during testing. Participants were instructed to extend their legs for five seconds.

The TUG procedure required participants to rise from their chairs, walk 3 meters at a self-selected speed, turn around, walk back, and sit down [20]. The test timing began with the word “go” and ended when the participant was seated.

The CS-30 assessed the functional strength of the lower extremities [21]. This test involved recording the number of repetitions (getting in and out of a chair) a participant could complete in 30 seconds. Higher scores on this test indicated greater physical function.

Sample size

This trial was designed to detect significant differences in the primary outcome (TSK) at a 5% significance level and 80% power, assuming an attrition rate of 12% [22]. Previous research in a similar population considered a 2-6-point difference in the TSK score to be clinically important [22]. Based on these considerations, it was calculated that 70 participants needed to be randomized.

Statistical analysis

Descriptive statistics, presented as mean (SD), were used to summarize the participant characteristics. Normality of data was performed using the Shapiro-Wilk test. The Mann-Whitney U test was used to compare TSK scores and other measurements between the two groups. A paired t-test was used to compare the pre- and post-intervention measurements of the participants. Cohen’s D effect sizes were computed as the mean difference relative to the pooled SD of baseline scores, with 0.2 considered a small effect, 0.5 considered a moderate effect, and 0.8 considered a large effect. As a subanalysis to identify factors associated with TSK, we performed correlations and multiple regression analyses. Spearman’s correlation was used to assess the association between TSK scores and other measurements. Additionally, a two-way repeated measures ANOVA was conducted to evaluate the interaction of interventions on TSK. All tests were performed with a significance level of p less than 0.05. All statistical analyses were performed using IBM SPSS Statistics for Windows, Version 24 (Released 2016; IBM Corp., Armonk, New York, United States).

## Results

**Figure 1:**
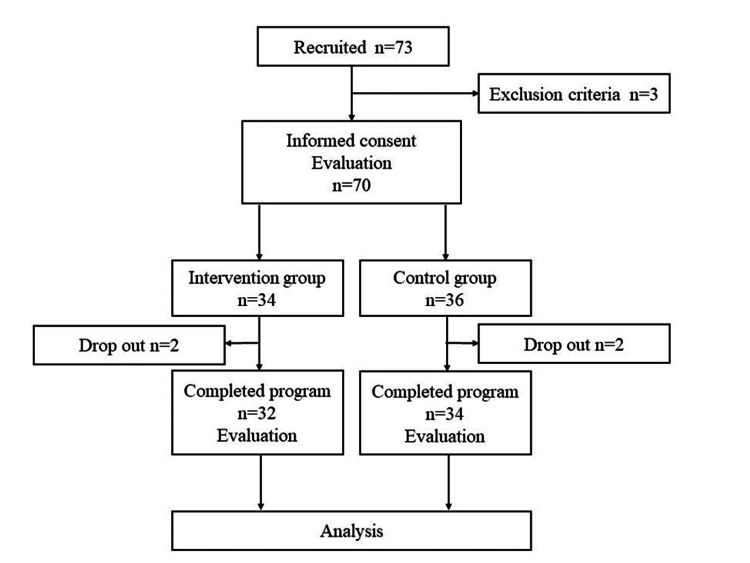
Flow diagram illustrating participant recruitment Seventy-three patients with chronic pain were recruited for this study, and written informed consent was obtained from 70 of them before their participation. The 70 participants were then categorized into an intervention group and a control group, with 32 and 34 participants in the intervention and control groups, respectively, completing the study.

Seventy-three patients with chronic pain were enrolled from April 2022 to March 2023; three were excluded based on the study’s exclusion criteria (Figure 1). The remaining 70 participants were divided into two groups. Two dropouts occurred in each of the intervention and control groups, resulting in 66 participants in the final analyses (intervention group, n=32; control group, n=34). The reason for dropout was the same in all cases: difficulty continuing outpatient treatment.

**Figure 2:**
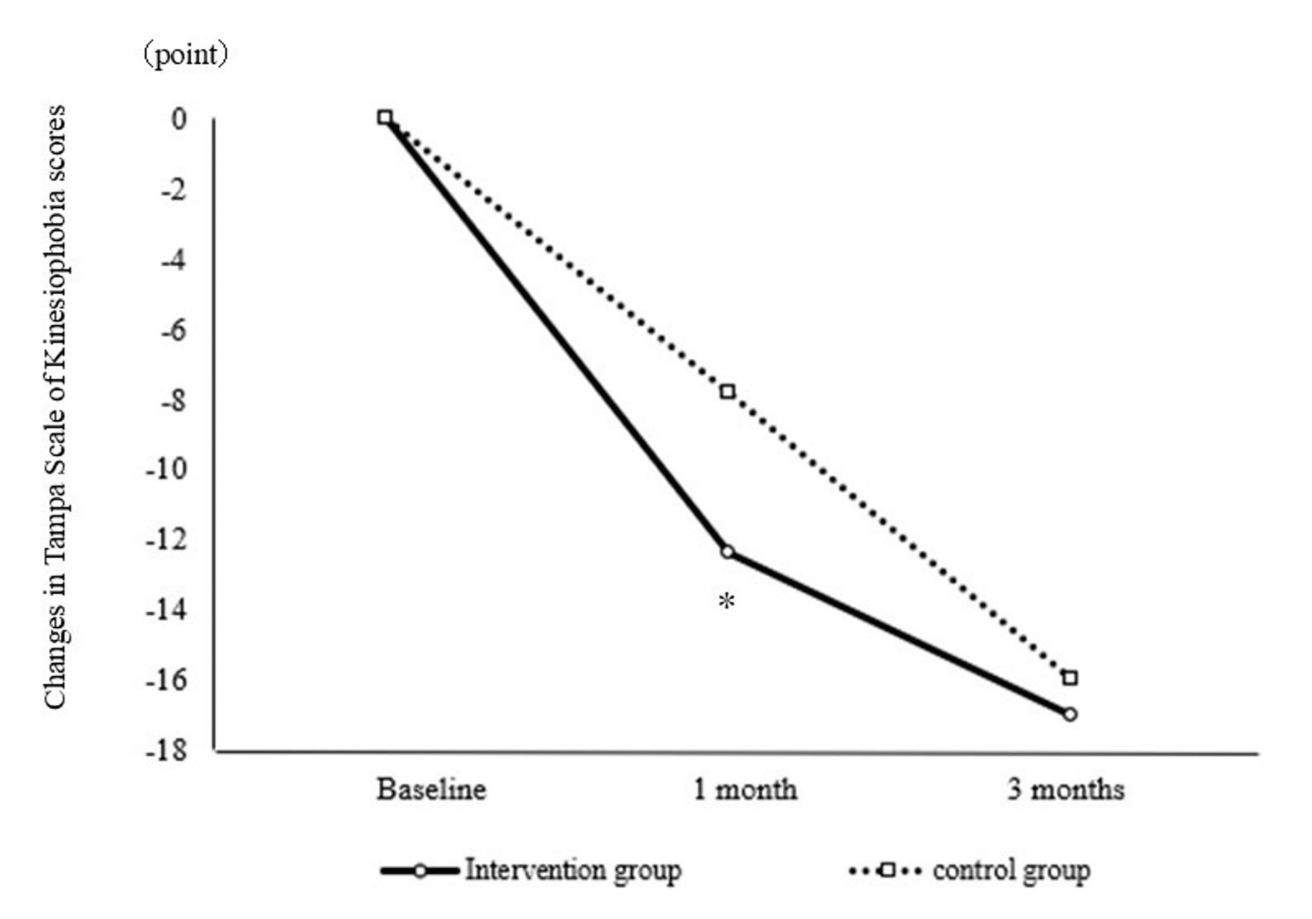
Comparison of changes in Tampa Scale of Kinesiophobia scores between the intervention and control groups A significant difference was observed when comparing the change in TSK scores between baseline and one-month post-intervention in the IG and CG. *p<0.05 TSK: Tampa Scale of Kinesiophobia; IG: intervention group; CG: control group

**Figure 3:**
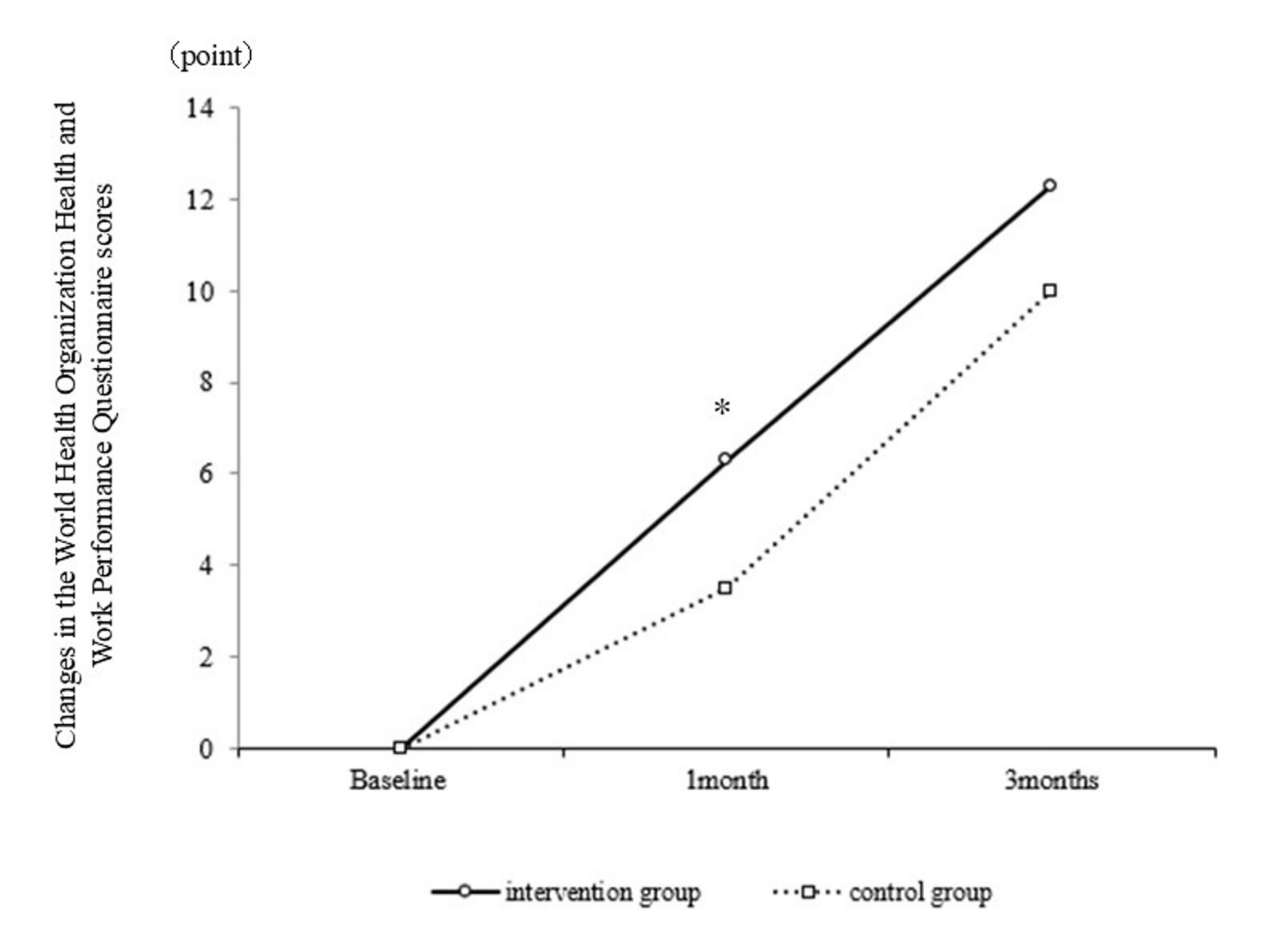
Comparison of changes in the World Health Organization Health and Work Performance Questionnaire scores between the intervention and control groups A significant difference was observed when comparing the change in WHO-HPQ scores between baseline and one-month post-intervention in the IG and CG. * p<0.05 WHO-HPQ: World Health Organization Health and Work Performance Questionnaire; IG: intervention group; CG: control group

**Figure 4:**
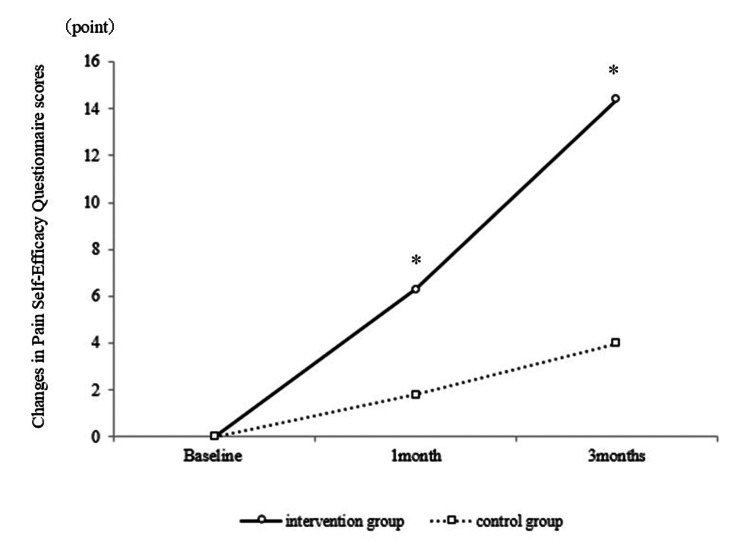
Comparison of changes in Pain Self-Efficacy Questionnaire scores between the intervention and control groups A significant difference was observed in the change in PSEQ scores between baseline and one-month post-intervention in the IG and CG. Furthermore, a significant difference was observed in the change in PSEQ scores between baseline and three months post-intervention in the IG and CG. * p<0.05 PSEQ: Pain Self-Efficacy Questionnaire; IG: intervention group; CG: control group

Participant characteristics are summarized in Table 1, presenting a comparison of outcomes between the intervention and control groups. No differences were observed between the groups at baseline. Table 2 presents a pre- and post-intervention comparison of participant scores. The intervention group demonstrated significant improvements in TSK and WHO-HPQ scores at one month (Figure 2) and three months (Figure 3) post-intervention, respectively. The intervention group also demonstrated significant improvements in PSEQ scores at one and three months post-intervention (Figure 4).

**Table 1:** Participant characteristics Values are reported as mean (SD) or number and percentage of participants. BMI: body mass index; NSAIDs: non-steroidal anti-inflammatory drugs

Characteristics	Intervention group (n=32）	Control group (n=34）	p-value
Male, n (%)	18 (56)	14 (44)	0.54
Age, years	52.6±10.9	56.8±14.1	0.74
Height, m	1.59±0.12	1.52±0.96	0.44
BMI, kg/ｍ^2^	21.8±5.5	24.7±4.5	0.55
Duration, month	38.2±18.2	42.3±21.5	0.71
Body part of pain, n (%)			
Neck	3 (9)	4 (12)	0.75
Shoulder	5 (16)	4 (12)	0.86
Elbow	1 (3)	1(3)	0.88
Lumbar	18 (56)	18 (53)	0.92
Knee	4 (13)	7 (20)	0.76
Ankle	1 (3)	0 (0)	0.88
Interventional treatment, n	17	21	0.69
Medication, n (%)			
NSAIDs	19 (59)	23 (68)	0.87
Acetaminophen	13 (41)	15 (44)	0.76
Weak opioids	11 (34)	8 (24)	0.82
Pregabalin	5 (16)	6 (18)	0.88
Others	26 (81)	28 (82)	0.84

**Table 2:** Comparison of the intervention and control groups Values are reported as mean (SD). ^†^p<0.05 (the results compared the baseline and one-month post-intervention characteristics between the intervention and control groups); *p<0.05, **p<0.01 (the results compared the baseline and three months post-intervention characteristics between the intervention and control groups). TSK: Tampa Scale of Kinesiophobia; WHO-HPQ: World Health Organization-Health and Work Performance Questionnaire; PSEQ: Pain Self-Efficacy Questionnaire; NRS: Numerical Rating Scale; HADS: Hospital Anxiety and Depression Scale; PCS: Pain Catastrophizing Scale; PDAS: Pain Disability Assessment Scale; IPAQ: International Physical Activity Questionnaire; EQ-5D-5L: EuroQol 5 Dimensions 5-Level; TUG: Timed Up and Go Test; CS-30: 30-s Chair Stand Test; ES: effect size

	Intervention group (IG)					Control group (CG)				
	Baseline	One month	ES	Three month	ES	Baseline	One month	ES	Three month	ES
TSK	37.2±4.3	24.8±3.1^†^	0.42	20.2±2.7**	0.59	38.2±3.2	30.4±3.9	0.32	22.2±3.2*	0.49
HPQ	66.2±14.1	72.5±5.2^†^	0.55	78.5±6.2*	0.43	62.3±9.9	65.8±5.9	0.21	72.3±5.9	0.32
PSEQ	22.3±4.1	28.6±3.1^†^	0.47	36.7±5.1**	0.39	26.1±5.8	27.9±3.8	0.22	30.1±3.8	0.24
NRS average	2.2±1.0	1.8±0.5	0.12	1.6±0.6	0.04	2.5±0.6	2.1±0.8	0.11	1.8±1.0	0.12
NRS minimum	0.7±0.8	0.5±0.7	0.21	0.4±1.2	0.09	0.9±0.9	1.0±0.8	0.09	1.2±1.0	0.02
NRS maximum	5.4±2.1	5.2±1.5	0.11	4.9±0.9	0.02	6.4±1.6	6.0±0.9	0.14	5.8±1.2	0.08
HADS total	18.6±4.5	14.5±3.3	0.31	11.8±2.8	0.23	16.2±5.2	14.0±2.7	0.16	13.3±3.2	0.05
HADS anxiety	10.2±1.8	7.3±1.3	0.20	6.3±1.0	0.13	9.5±2.3	7.1±0.9	0.13	7.2±1.2	0.11
HADS Depression	8.4±1.2	6.3±1.6	0.18	5.5±1.2	0.11	6.7±1.8	6.9±1.1	0.11	6.1±1.1	0.02
PCS total	28.7±5.2	19.5±3.1^†^	0.59	16.2±2.3**	0.62	27.0±4.3	21.5±4.1	0.24	18.0±3.3*	0.33
PCS rumination	13.5±3.1	9.3±1.6^†^	0.49	8.3±1.9*	0.44	14.1±2.1	11.1±1.9	0.45	9.2±2.6*	0.43
PCS helplessness	9.3±2.1	6.8±1.5	0.33	5.5±2.0	0.31	8.7±4.1	7.2±3.2	0.12	6.2±2.1	0.14
PCS magnification	5.9±1.6	3.4±0.7	0.19	2.4±0.8	0.21	4.2±0.6	3.2±0.8	0.21	3.0±0.6	0.16
IPAQ (MET-m/w)	855.5±633.1	1788.4±702.4^†^	0.54	2238.5±771.9**	0.70	688.5±361.0	1233.8±627.4	0.14	1678.5±421.7	0.19
EQ-5D-5L	0.76±0.12	0.79±0.08	0.03	0.80±0.15	0.06	0.73±0.21	0.78±0.28	0.14	0.82±0.31	0.01
Quadriceps muscle strength (kgf/kg)	0.41±0.03	0.43±0.07	0.04	0.47±0.10	0.04	0.38±0.05	0.39±0.06	0.21	0.41±0.08	0.01
TUG (second)	7.7±3.8	7.4±1.5	0.04	7.1±1.8	0.02	8.2±2.2	7.9±1.9	0.24	7.8±2.2	0.20
CS-30 (times)	15.8±6.3	17.2±5.5	0.23	19.3±4.5*	0.35	16.3±6.9	17.1±5.9	0.29	18.2±5.9	0.21

Additionally, significant improvements were observed in TSK scores, presenteeism, PCS, IPAQ, PSEQ, and CS-30 scores after three months in the intervention group (p<0.05). The control group also demonstrated significant improvements in TSK and PCS scores at three months post-intervention (p<0.05). Significant differences in HADS, PDAS, PSEQ, and CS-30 score changes were observed between the groups after the intervention (p<0.05; Table 3).

**Table 3:** Comparison of changes in the intervention and control groups Values are reported as mean (SD). *p<0.05 TSK: Tampa Scale of Kinesiophobia; WHO-HPQ: World Health Organization Health and Work Performance Questionnaire; PSEQ: Pain Self-Efficacy Questionnaire; NRS: Numerical Rating Scale; HADS: Hospital Anxiety and Depression Scale; PCS: Pain Catastrophizing Scale; PDAS: Pain Disability Assessment Scale; IPAQ: International Physical Activity Questionnaire; EQ-5D-5L: EuroQOL 5 Dimensions 5-Level; TUG: Timed Up and Go Test; CS-30: 30-s Chair Stand test.

	Baseline and one month			Baseline and three month		
	IG change	CG change	p-value	IG change	CG change	p-value
TSK	-12.4±2.1	-7.8±1.4	0.04*	-17.0±3.8	-16.0±3.1	0.69
HPQ	6.3±2.3	3.5±2.3	0.03*	12.3±2.3	10.0±2.1	0.34
PSEQ	6.3±2.3	1.8±2.3	0.04*	14.4±2.9	4.0±1.6	0.02*
NRS average	-0.4±0.1	-0.4±0.2	0.88	-0.6±0.2	-0.7±0.7	0.77
NRS minimum	-0.2±0.05	0.1±0.03	0.76	-0.3±0.3	0.3±0.4	0.83
NRS maximum	-0.2±0.1	-0.4±0.1	0.65	-0.5±0.1	-0.6±0.2	0.76
HADS total	-4.1±1.7	-2.2±0.7	0.12	-6.8±1.0	-2.9±1.2	0.04*
HADS anxiety	-2.9±1.2	-2.4±1.1	0.33	-3.9±0.3	-2.3±0.1	0.32
HADS Depression	-2.1±0.09	0.2±0.1	0.03*	-2.9±0.04	-0.6±0.08	0.03*
PCS total	-9.2±2.3	-5.5±2.4	0.08	-12.5±2.9	-9.0±3.1	0.17
PCS rumination	-4.2±0.5	-3.0±1.2	0.22	-5.2±0.4	-4.9±0.2	0.22
PCS helplessness	-2.5±0.9	-1.5±1.1	0.21	-3.8±0.8	-2.5±0.6	0.12
PCS magnification	-2.5±0.5	-1.0±0.2	0.21	-3.5±0.9	-1.2±0.1	0.21
IPAQ (MET-m/w)	932.9±334.6	545.3±455.9	0.43	1383.0±411.2	990.0±285.1	0.70
EQ-5D-5L	0.03±0.03	0.05±0.02	0.23	0.04±0.01	0.09±0.02	0.32
Quadriceps muscle strength (kgf/kg)	0.02±0.03	0.01±0.04	0.63	0.06±0.02	0.03±0.01	0.69
TUG (second)	-0.3±0.2	-0.3±0.09	0.77	-0.6±0.1	-0.39±0.07	0.15
CS-30 (times)	1.4±0.6	0.8±0.8	0.19	3.5±1.3	1.9±1.3	0.03*

Table 4 shows the relationship between changes in TSK scores, self-reported measures, and functional performance. Multivariate regression analysis at one-month post-intervention revealed that the PCS, PSEQ, IPAQ, and intervention were significantly associated with TSK scores (Table 5). The goodness of fit for the logistic model was tested, with the coefficient of determination (R^2^) reported as 0.62 (p <0.01). Multivariate logistic regression analysis at three months post-intervention revealed that the PCS, PSEQ, HADS, and intervention were significantly associated with TSK scores (Table 6). The goodness of fit for the logistic model was tested, with the coefficient of determination (R^2^) reported as 0.49 (p<0.01). Table 7 shows the results of a two-way repeated measures ANOVA on the change in TSK score after intervention. There was an interaction on the improvement of TSK.

**Table 4:** Relationship between changes in TSK scores, self-report measures, and functional performance *p<0.05, **p<0.01 WHO-HPQ: World Health Organization Health and Work Performance Questionnaire; PSEQ: Pain Self-Efficacy Questionnaire; NRS: Numerical Rating Scale; HADS: Hospital Anxiety and Depression Scale; PCS: Pain Catastrophizing Scale; PDAS: Pain Disability Assessment Scale; IPAQ: International Physical Activity Questionnaire; EQ-5D-5: EuroQOL 5 Dimensions 5-Level; TUG: Timed Up and Go Test; CS-30: 30-s Chair Stand test

	One month		Three months	
	Correlation coefficient	p-value	Correlation coefficient	p-value
ΔHPQ	-0.42	0.02*	-0.33	0.14
ΔPSEQ	-0.46	0.02*	-0.43	0.03*
ΔNRS average	0.11	0.33	0.23	0.55
ΔNRS minimum	0.23	0.29	0.19	0.62
ΔNRS maximum	0.33	0.77	0.41	0.02*
ΔHADS total	0.28	0.56	0.41	0.03*
ΔHADS anxiety	0.14	0.23	0.29	0.13
ΔHADS Depression	0.19	0.21	0.38	0.08
ΔPCS total	0.39	0.02*	0.55	<0.01**
ΔPCS rumination	0.25	0.04*	0.34	0.04*
ΔPCS helplessness	0.33	0.18	0.32	0.54
ΔPCS magnification	0.30	0.21	0.29	0.33
ΔIPAQ (MET-m/w)	-0.43	0.02*	-0.56	0.02*
ΔEQ-5D-5L	-0.13	0.74	-0.21	0.65
ΔQuadriceps muscle strength (kgf/kg)	-0.08	0.73	-0.22	0.66
ΔTUG (second)	0.28	0.53	0.11	0.55
ΔCS-30 (times)	-0.37	0.43	-0.39	<0.01**

**Table 5:** Multiple regression analysis of changes in TSK scores at one-month post-intervention and explanatory variables *p<0.05, **p<0.01 PCS: Pain Catastrophizing Scale; PSEQ: Pain Self-Efficacy Questionnaire; IPAQ: International Physical Activity Questionnaire

	β	t	p-value	95% CI	
				Lower	Upper
Constant		2.88	0.04*	2.25	6.44
ΔPCS total	0.43	2.51	<0.01**	0.34	1.11
ΔPSEQ	0.3	1.22	0.02*	0.83	1.43
ΔIPAQ	0.22	0.88	0.02*	0.02	1.21
Intervention	0.32	1.43	0.01*	0.09	2.45
					R^2^=0.62

**Table 6:** Multiple regression analysis of changes in TSK scores at three months post-intervention and explanatory variables *p<0.05 PCS: Pain Catastrophizing Scale; PSEQ: Pain Self-Efficacy Questionnaire; HADS: Hospital Anxiety and Depression Scale

	β	t	p-value	95% CI	
				Lower	Upper
		3.58	0.03*	1.12	4.55
ΔPCS total	0.29	2.01	0.01*	0.11	0.89
ΔPSEQ	0.32	3.22	0.02*	0.23	1.44
ΔHADS total	0.51	2.22	0.02*	0.55	1.24
Intervention	0.19	1.12	0.04*	0.09	2.12
					R^2^=0.49

**Table 7:** Two-way repeated measures ANOVA on the change in TSK score after intervention Values are reported as mean (SD). *p<0.05 TSK: Tampa Scale of Kinesiophobia

	Intervention group (IG)		Control group (CG)			
	Change of baseline and one month	Change of baseline and three month	Change of baseline and one month	Change of baseline and three month	F-value	p-value
TSK	-12.4±2.1	-17.0±3.8	-7.8±1.4	-16.0±3.1	7.52	0.03*

## Discussion

This study revealed two significant findings: (i) combining physical therapy with VR effectively reduced pain-related fear of movement in patients at an early stage, and (ii) changes in kinesiophobia scores in patients with chronic pain were associated with changes in pain catastrophizing, self-efficacy, and VR intervention.

Pain-related fear of movement affects 51-72% of patients with chronic pain [23]. It is a component of the fear-avoidance model [1], which suggests that when a painful event is perceived as threatening, it can lead to catastrophic beliefs that movement and physical activity can exacerbate pain and injury. Previous studies have shown that catastrophic thoughts can occur when patients experience pain [24], worsening bodily dysfunction, and increasing pain [2]. Breaking this cycle of movement avoidance and the resulting decline in functional ability caused by pain-related fear of movement is important to improve patients’ ADL and QOL.

TSK scores in the intervention group significantly improved at one-month post-intervention compared to those in the control group (Table 2 and Figure 2). Combining VR with physical therapy effectively mitigated the excessive pain-related fear of movement. In this study, VR provided an exercise experience without physical pain for patients with chronic pain. This was intended to create a calming, distractive-filled environment, thereby reducing the perceived threat of kinesiophobia. Furthermore, it is possible that there was a reduction in central sensitization [25]. We believe that reducing pain-related fear of movement made exercise therapy even more effective. Previous studies have shown that exercise activates descending pain modulation, leading to pain reduction [26]. However, pain perception has also been reduced by imagining exercise without actually engaging in physical activity [6]. It was suggested that separating physical exercise from pain sensations could reduce pain-related fear of movement. Previous studies have demonstrated the promising efficacy of VR interventions for patients with chronic neck pain, either when used alone or in conjunction with exercise [27]. In this study, an early reduction in pain-related fear of movement was achieved despite the diverse chronic pain conditions among patients.

TSK scores in both groups improved significantly at three months post-intervention (Table 2). Specifically, the intervention group’s TSK scores improved over three months with ongoing intervention. The combination of physical therapy and VR reduced pain-related fear of movement in the early stages. This suggests that while long-term exercise may reduce pain-related fear of movement, the benefits can be realized early using a combination of VR and exercise. Previous research suggests that the therapeutic mechanisms of VR for chronic low back pain are unclear but may include distraction, neuromodulation of body perception, and graded exposure therapy [5]. VR is thought to separate physical activity from the experience of pain, reduce catastrophic thinking, and increase self-efficacy; however, continued use of VR is considered important.

Multivariate analysis revealed that ΔPCS, ΔPSEQ, and intervention were common elements of ΔTSK at one and three months post-intervention (Tables 5-6). These findings suggest that combining physical therapy with VR and improved PCS and PSEQ scores contributed to enhanced TSK scores. Furthermore, a two-way repeated measures ANOVA of variance revealed an interaction in the change in TSK, demonstrating the usefulness of combined VR and physical therapy intervention. Visual and auditory stimulation may be effective in managing pain. The intervention group in this study experienced an immersive VR intervention that involved viewing a natural landscape. Previous research has suggested that immersive VR is more effective than non-immersive VR. Immersive VR and game-like tasks are thought to reduce pain-related fear of movement in patients with chronic pain [28]. Patients with pain-related fear of movement often believe that avoiding movement is an appropriate response, leading to detrimental behaviors and decreased overall functional ability. This behavior aggravates the disorder and increases pain sensation [2]. Early intervention is essential as prolonged pain can cause central sensitization [25]. Therefore, for patients with a strong pain-related fear of movement, we think it is important to first use VR to give them the illusion that exercise will not worsen their pain.

VR could be beneficial in managing pain-related fear of movement by creating the illusion that exercise does not cause pain. However, caution should be taken into account regarding potential adverse effects on clinical practice, such as discomfort, motion sickness, and lack of immersion. This was not observed in participants in the present study. As a proposed future experimental approach, it may be possible to clarify VR's mechanisms, for example, by measuring cerebral blood flow and conducting quantitative sensory tests.

This study had a few limitations. First, the multifaceted pain evaluation was conducted using a questionnaire, which may have limited the accuracy of judgments due to the subjective nature of recall. Second, the results may not be generalizable to all patients with chronic pain, as participants were specifically those who underwent physical therapy, potentially introducing selection bias. Third, the findings were obtained from a single pain clinic, making it unclear whether they can be generalized to patients in other hospitals or facilities.

## Conclusions

Our findings suggest that chronic pain patients who receive a combined VR and physical therapy intervention may experience reduced pain-related fear of movement one month later than patients who receive a physical therapy-only intervention. Considering future research directions, we intend to categorize pain by intensity and location, implement VR interventions that incorporate movement, and include sham intervention arms in our research.
